# The role of optical coherence tomography angiography in fundus vascular abnormalities

**DOI:** 10.1186/s12886-016-0277-2

**Published:** 2016-07-13

**Authors:** Shanshan Yu, Jing Lu, Di Cao, Ruyuan Liu, Bingqian Liu, Tao Li, Yan Luo, Lin Lu

**Affiliations:** State Key Laboratory of Ophthalmology, Zhongshan Image Reading Center, Zhongshan Ophthalmic Center, Sun Yat-Sen University, Guangzhou, 510060 China

**Keywords:** Optical coherence tomography angiography, Fundus vascular abnormalities, CNV, DR, BRVO, PCV

## Abstract

**Background:**

To evaluate the role of optical coherence tomography angiography (OCTA) in observation of fundus vascular abnormalities.

**Methods:**

Patients (*n* = 50, 10 in each group) with fundus disorders including branch retinal vein occlusion (BRVO), non-proliferative diabetic retinopathy (NPDR), proliferative diabetic retinopathy (PDR), exudative age-related macular degeneration (AMD), and polypoidal choroidal vasculopathy (PCV) were examined. They underwent imaging of OCTA and fluorescein angiography/indocyanine green angiography. The split-spectrum amplitude-decorrelation angiography algorithm was employed to obtain angiography within a 6 × 6 mm scanning area at the posterior retina. Segmentation algorithm was used to obtain 2-dimensional images from arbitrary layers. The OCTA features were analyzed and compared with the findings of conventional angiography. The contralateral eyes of the patients with BRVO and the eyes of 20 healthy volunteers served as controls.

**Results:**

OCTA showed precise images of normal and abnormal vasculature in the posterior retina and choroid by the given layers. Vascular abnormalities such as enlarged foveal avascular zone (FAZ), non-perfusion area of retina, microaneurysm, retinal neovascularization, choroidal neovascularization (CNV), branching vascular network and polypoidal lesions in choroid were clearly displayed by OCTA.

**Conclusions:**

OCTA provided a better projection of vascular pathologies of the posterior retina and choroid and could determine the precise location of the vascular lesion. The noninvasive OCTA can benefit the diagnosis of vascular abnormalities in the posterior retina and choroid.

## Background

In the past four decades, fundus angiography including fluorescein angiography (FA) and indocyanine green angiography (ICGA) have important roles in diagnosing and evaluating retinal vascular diseases in clinical practice. However, both FA and ICGA are invasive, requiring intravenous dye injection. These examinations can cause side effects as extravasation, nausea [1], vasovagal reaction [2], and anaphylaxis [3, 4]. It is current practice to avoid FA and ICGA in people allergic to dye or pregnant. Other previous techniques, such as laser Doppler velocimetry [5] and laser speckle phenomenon [6], have been applied to detect blood velocity and flow in retinal and choroidal vascular diseases [7, 8]. However, laser Doppler velocimetry fails to reconstruct two dimensional flow images [5], and both of these two methods merely detect blood flow in large retinal and choroidal vessels. Therefore, new ways of detecting retinochoroidal vasculature abnormalities are necessary.

The split-spectrum amplitude-decorrelation angiography (SSADA) algorithm, recently developed by David Huang’s group [9, 10], has revolutionized the way to observe retinochoroidal vascular structure. SSADA is based on detecting the reflectance amplitude variation of blood flow over time to distinguish vessels from static tissue. An intrinsic contrast between static and non-static tissue is visualized without dye injection by calculating the decorrelation of signal amplitude from consecutive B-scans [11]. Decorrelation generated by bulk eye motion and orbital pulsation are removed by registering four orthogonal raster scanned volumes, from whom the flow data are weighted and merged [12]. Segmentation algorithm produces transversal slices of the retinal and choroidal layer at any depth. Together, these two techniques provide a new technology, which can transversally scan retina and choroid at various levels with distinct vascular conformation. Some studies have investigated various retinal and choroidal diseases with optical coherence tomography angiography (OCTA) [13–15], however, no study offers an overview of different sorts of fundus vascular abnormalities that are visualized by OCTA. Therefore, the purpose of this study was to investigate the role of the non-invasive OCTA in detecting the vascular disturbances of retina and choroid.

## Methods

We studied patients (*n* = 50, 10 in each group) with branch retinal vein occlusion (BRVO), non-proliferative diabetic retinopathy (NPDR), proliferative diabetic retinopathy (PDR), exudative age-related macular degeneration (exudative AMD), and polypoidal choroidal vasculopathy (PCV) at the outpatient clinic from November 2014 to July 2015. The contralateral eyes of the patients with BRVO and the eyes of twenty healthy volunteers with no medical history of ocular or systemic diseases served as controls. Because only with the good fixation of the patients can OCT angiography be performed with fair quality, the patients enrolled all had the best corrected visual acuity (LogMAR) no less than 1.0.

Fundus FA and ICGA were performed by HRA-2 (Heidelberg Engineering, Germany). OCTA images was captured by the RTVue XR 100 Avanti OCT (Optovue, Inc, USA). The spectral-domain OCT was operated at an axial scan speed of 70 kHz (26,000 A-scans/s) with a wavelength of 840 nm. A transverse resolution of 8 μm and axial resolution of 5 μm was achieved. All patients went through four different scanning patterns: the cross line scan, the 3D Widefield motion correction technology (MCT), the 3D retina scan, and the angio-retina scan. The cross line scan produced a cross-sectional OCT image with a length of 10 mm in both vertical and horizontal directions, which revealed the structure of the lesion. The 3D Widefield MCT scan was used for the reference of the lesion location, providing a 12 × 9 mm 3D SLO-like image of an approximately 40°fundus scope. The 3D retina scan (7 × 7 mm) produced a color-coded thickness map, which provided an overview of the retinal thickness. Although the images from these three scanning modes were not shown in this paper, these modes were very important for the clinician to acquire a comprehensive picture of the studied vasculopathy. Another scanning pattern used in this study was the angio-retina scan, which captured a 6 × 6 mm area of the posterior pole and displayed the retinal and choroidal vascular networks in different depth. Usually, the angio-retina scan centered on the fovea or focused on the targeted vasculopathy. The preset layers of the superficial vascular plexus, the deep vascular plexus, the outer retina, and the choroid capillary were analyzed. When automated segmentation and its relative depth in the retina and choroid were inaccurate or incorrect due to the influence of local lesions, manual adjustment was applied.

## Results

The profiles of the study subjects were listed in Table 1. Fundus vasculature was illustrated by OCTA at different retinal and choroidal layers: the superficial vascular plexus, the deep vascular plexus, the outer retina, and the choroid capillary. The superficial vascular plexus was defined from the internal limiting membrane (ILM) offset of 3 μm to the inner plexiform layer (IPL) offset of 15 μm. The deep vascular plexus was set from the IPL offset of 15 μm to the IPL offset of 70 μm. The outer retina was set from the IPL offset of 70 μm to the retinal pigment epithelium (RPE) offset of 30 μm. The choroid capillary layer visualized not only the choriocapillaris, but also the medium and large blood vessels. The level of the exhibited OCT angiogram was indicated by double lines in B scan OCT image. Normal subjects were set as controls, providing the reference for the abnormal signs described below.

**Table 1:** The profiles of the study subjects

	BRVO (*n* = 10)	NPDR (*n* = 10)	PDR (*n* = 10)	Exudative AMD (*n* = 10)	PCV (*n* = 10)	Control (*n* = 20)
Age (years)	49.4 ± 8.1 (35–63)	54.0 ± 7.8 (41–65)	56.7 ± 6.5 (45–68)	62.6 ± 6.6 (53–73)	53.8 ± 7.7 (40–64)	50.2 ± 6.6 (43–60)
Male/female (*n*)	5/5	6/4	4/6	6/4	7/3	10/10
BCVA (LogMAR)	0.3 ± 0.2 (0.1–0.5) (Contralateral eyes: 0.0 ± 0.0 (0.0–0.1))	0.0 ± 0.1 (−0.1–0.2)	0.4 ± 0.2 (0.0–0.7)	0.7 ± 0.2 (0.4–1.0)	0.7 ± 0.2 (0.5–1.0)	0.0 ± 0.0 (−0.1–0.0)

*BRVO* branch retinal vein occlusion, *NPDR* non-proliferative diabetic retinopathy, *PDR* proliferative diabetic retinopathy, exudative *AMD* exudative age-related macular degeneration, *PCV* polypoidal choroidal vasculopathy

**Fig. 1:**
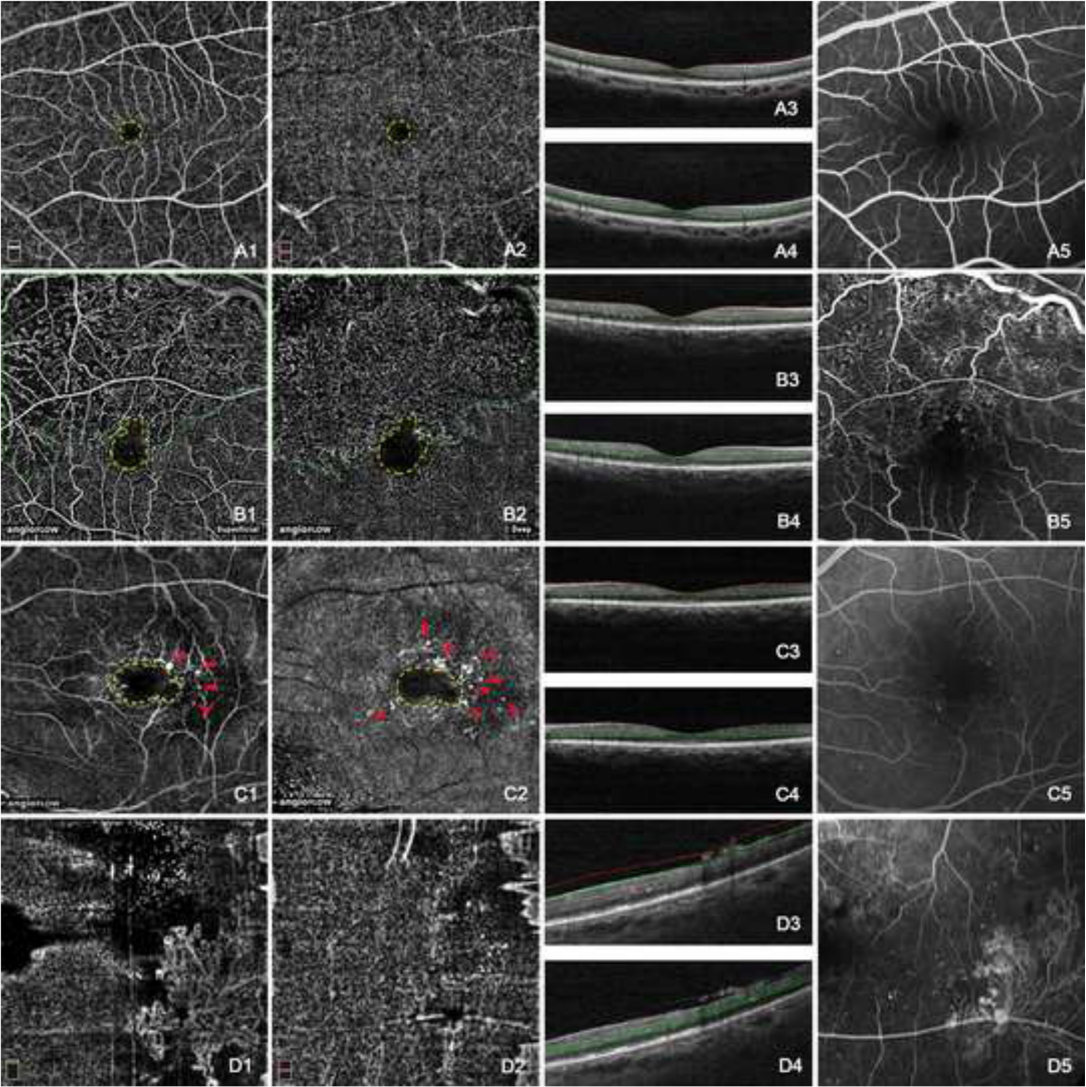
Normal retinal vasculature and retinal vascular abnormalities showing by OCT angiograms. The normal appearance of the superficial (*a1*) and deep (*a2*) vascular plexus showed an intact fovea avascular zone (FAZ) surrounding by distinct and homogeneous web-like retinal vascular networks. Large retinal vessels in the superficial vascular plexus were reflected onto the deep vascular plexus (*a2*). The early phase of normal FA image was shown in (*a5*). FAZ was enlarged and extended superiorly (*b1*, *b2*) and temporally (*c1*, *c2*). Both the superficial and the deep vascular plexuses were affected. The nonperfusion areas were revealed superotemporally to the fovea and the vascular meshes became sparse and irregular with the tortuous and dilated residual capillaries (*b1*, *b2*). Microaneurysms showed as hyperreflective dots around the FAZ in both the superficial vascular plexus (*c1*) and the deep vascular plexus (*c2*). Retinal neovascularization was mainly located prior to the superficial vascular plexus (*d1*), distinctively delineated as flower-like branching loops. The nonperfusion areas in FA image (*b5*) seemed less profound than those in OCT angiograms. Microaneurysms were better illustrated in FA image (*c5*). The enlarged FAZs (*b5*, *c5*) and retinal neovascularization (*d5*) in FA images appeared identical in shape with those in corresponding OCT angiograms. The *double lines* in B scan OCT images of *a3*, *a4*, *b3*, *b4*, *c3*, *c4*, *d3*, and *d4* indicated the layers exhibited in *a1*, *a2*, *b1*, *b2*, *c1*, *c2*, *d1*, and *d2* respectively. (*Yellow dashed line*: FAZ; *Green dashed line*: retinal nonperfusion area; *Red arrowhead*: microaneurysm; *Red dashed line*: retinal neovascularization)

The normal appearances of superficial and deep vascular plexuses in OCT angiogram were demonstrated in Fig. 1*a1*-*a4*. Fovea avascular zone (FAZ) was round and intact with a well-demarcated border in both plexuses. At the level of the superficial vascular plexus, large retinal vessels and delicate retinal vascular networks were distinct against the black background. At the level of the deep vascular plexus, normal capillary beds showed a homogeneous grayish texture, with “mirror image” of the large retinal vessels from the layer above. Retinal vascular abnormalities such as enlargement of FAZ, vascular network attenuation, retinal nonperfusion, microaneurysm, and retinal neovascularization were demonstrated in Fig. 1 at both the superficial and the deep vascular plexuses. Enlargement of FAZ was showed in the cases of BRVO (Fig. 1*b1*-*b4*) and NPDR (Fig. 1*c1*-*c4*). The extended FAZ appeared as well demarcated irregular circles with hyporeflection. Retinal nonperfusion and vascular network attenuation were revealed in the cases of BRVO (Fig. 1*b1*-*b4*) and PDR (Fig. 1*d1*-*d4*). The hyporeflective nonperfusion areas were separated by the sparse vascular meshes, of which the veins and capillaries were dilated and tortuous. Microaneurysms were showed in the case of NPDR (Fig. 1*c1*-*c4*) around the FAZ as hyperreflective dots or pinpoints. Retinal neovascularization depicted in the case of PDR (Fig. 1*d1*-*d4*) had a tangled vasculature in a flower-like shape above retina. As in FA images, the enlarged FAZs and retinal neovascularization appeared identical in shape with those in OCT angiograms; the nonperfusion areas seemed less profound than those in OCT angiograms due to the incomplete overlap of the nonperfusion areas in the superficial vascular plexuses and the deep vascular plexuses; microaneurysms were better illustrated.

**Fig. 2:**
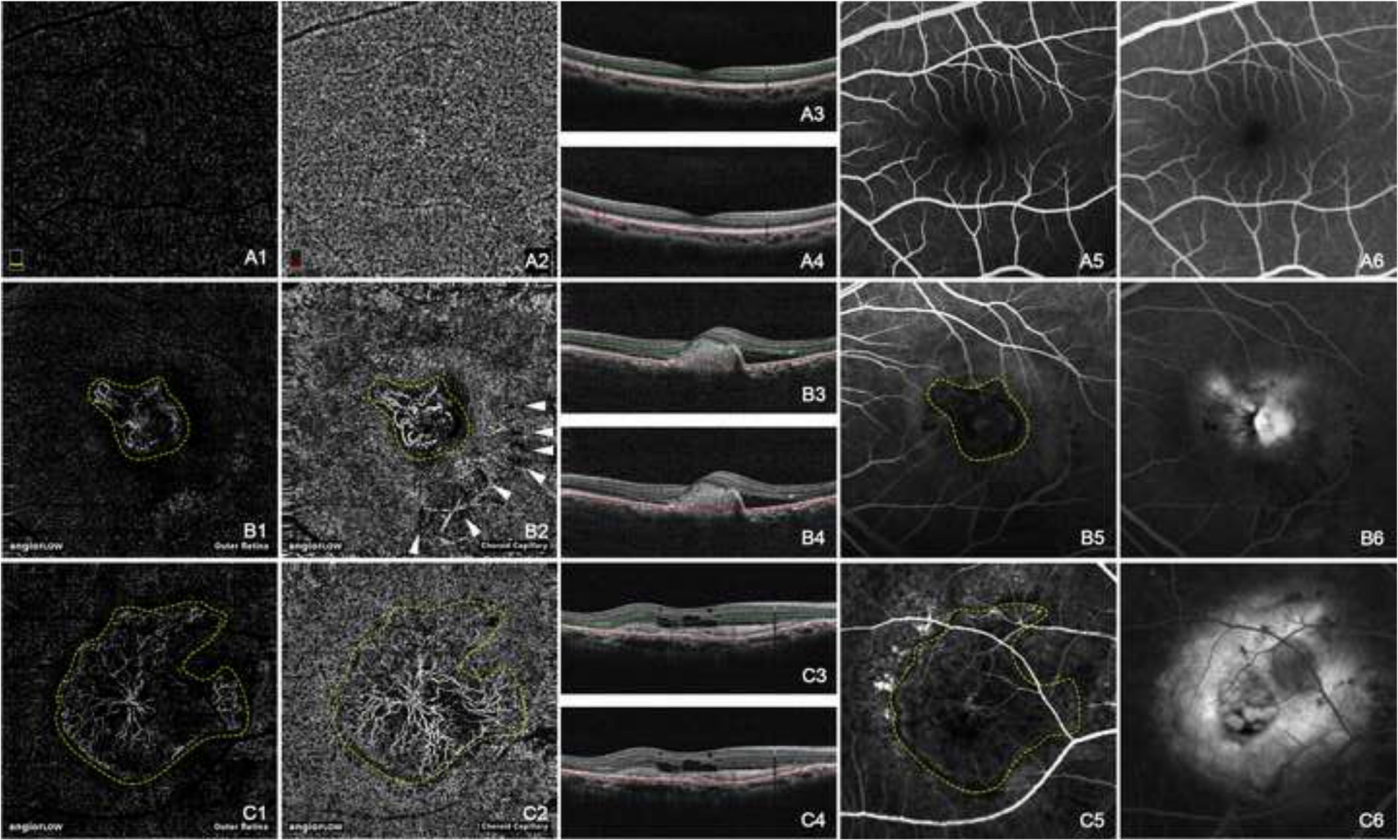
Normal choroidal vasculature and choroidal neovascularization (CNV) showing by OCT angiograms. The normal appearance of the outer retina showed in absence of blood flow (*a1*). The normal appearance of the choroid capillary showed with homogeneous grayish capillary beds (*a2*). The early phase and the late phase of normal FA images were shown in (*a5*) and (*a6*) respectively. Hyperreflective CNVs were demonstrated at both the outer retina level (*b1*, *c1*) and the choroid capillary level (*b2*, *c2*). The configurations of these CNV lesions were strikingly identical with those in the early phase of FA (*b5*, *c5*). CNV was in a globular aspect in (*b1*) and (*b2*), while in a fan-like shape with a feeder vessel in (*c1*) and (*c2*). The hyporeflective speckles (*white arrowheads*, *b2*) regarding the hard exudates were “mirror images” projected from the deep retinal plexus layer (not shown). The early phase of FA (*b5*, *c5*) revealed hyperfluorescent CNVs, while the late phase of FA (*b6*, *c6*) showed leakage and edema caused by CNV. The *double lines* in B scan OCT images of *a3*, *a4*, *b3*, *b4*, *c3*, and *c4* indicated the layers exhibited in *a1*, *a2*, *b1*, *b2*, *c1*, and *c2* respectively. (*Yellow dashed line*: CNV; *White arrow head*: “mirror image” of hard exudate)

**Fig. 3:**
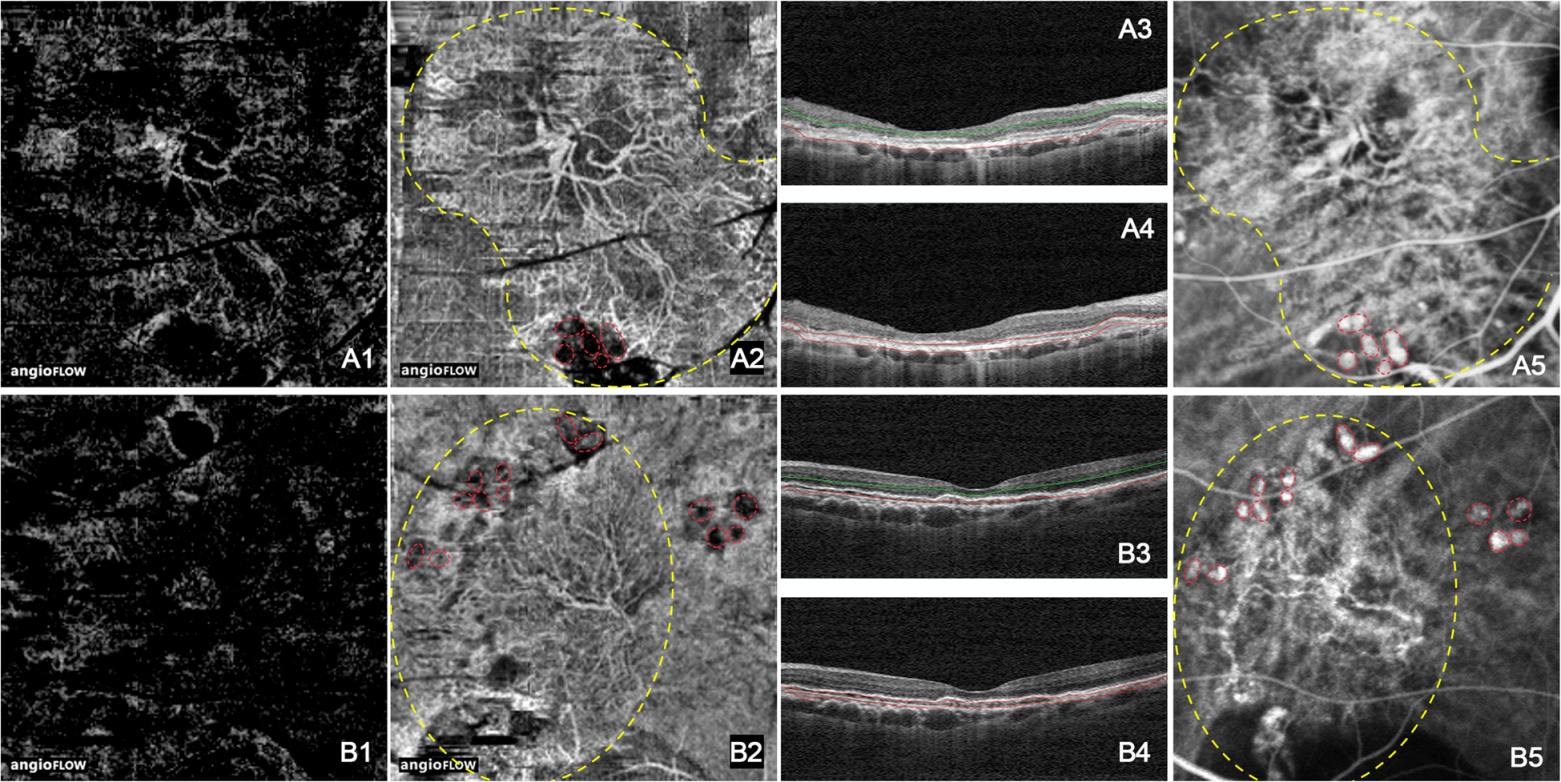
Branching vascular networks (BVNs) and polypoidal lesions showing by OCT angiograms. The hyperreflective BVNs were primarily located at the choroid capillary level (*a2*, *b2*). BVNs were faint or absolutely fade out at the outer retinal level (*a1*, *b1*). The polypoidal lesion was a cluster of hyperreflective, medium or hyporeflective spots (*red dashed circles* in *a2* and *b2*). On ICGA images, polypoidal lesions showed as clusters of hyperfluorescent spots (*red dashed circles* in *a5* and *b5*) in connection to the ends of BVNs. The *double lines* in B scan OCT images of *a3*, *a4*, *b3*, and *b4* indicated the layers exhibited in *a1*, *a2*, *b1*, and *b2* respectively. (*Yellow dashed line*: BVN; *Red dashed line*: polypoidal lesion)

The normal appearances of the outer retina and the choroid capillary in OCT angiogram were demonstrated in Fig. 2*a1*-*a4*. The outer retinal layer had no signal of blood flow. The choroid capillary layer showed homogeneous grayish capillary beds without any nonperfusion areas. The “mirror image” of the large retinal vessels from the superficial vascular plexus was also visible at this layer. Choroidal vascular abnormalities such as choroidal neovascularization (CNV), branching vascular networks (BVNs) and polypoidal lesions were demonstrated in Figs. 2 and 3 at both the outer retina layer and the choroid capillary layer. CNV was demonstrated in the cases of exudative AMD (Fig. 2*b1*-*c6*). In the OCT angiogram, CNV was distinct at both the choroid capillary level and the outer retina level, occupying a rather thin slab adjacent to the Bruch’s membrane. It could have a fan-like appearance with a feeder vessel (Fig. 2*c1*-*c6*) or a globular aspect as if it was wrapped by an invisible capsule (Fig. 2*b1*-*b6*). Hard exudates that accompany the CNV lesion displayed at the outer retina layer were projected from those in the deep vascular plexus (Fig. 2*b2*). In FA images, CNV was hyperfluorescent at the early phase and leaking at the late phase. Hard exudates and hemorrhages could be clearly revealed (Fig. 2*b5* and *b6*). BVNs and polypoidal lesions were demonstrated in the cases of PCV (Fig. 3). In the OCT angiogram, the BVNs were best seen between the RPE and choroid capillary. The polypoidal lesion was revealed as a cluster of hyperreflective, medium or hyporeflective spots (red dashed circles in Fig. 3*a2* and *b2*) when scrolling through different depths from RPE to choroid capillary. BVN was better displayed in OCT angiograms than in ICGA image, while polyps were not defined very well in OCT angiograms.

## Discussion

OCT is an effective ‘optical ultrasound’, providing cross-sectional images by capturing reflections from tissue. With the consecutive development of time domain OCT, frequency domain OCT, spectral domain OCT, and swept source OCT, images of retinal structure are more and more clearly demonstrated now. However, it offers no clues about retinal and choroidal vasculature and fails to give an overview of the affected vascular networks. The newly developed OCTA uses spectral domain OCT or swept source OCT as a basis and revolutionarily adds SSADA and segmentation algorithms to it. Compared to the normal control, retinal and choroidal vasculopathies in OCTA are identified by the presence of flow in normally non-vascular layers or the absence and disturbance of flow in normally vascular layers. OCTA has a range of advantages that make it an alternative imaging modality in detecting choroidal and retinal circulation. First, its noninvasive nature allows frequent assessments of choroid and retina in the follow-up visits. Second, its 3D nature facilitates the reconstruction and evaluation of retinal and choroidal vasculatures at various depths. The precise location, accurate anatomic feature, and detailed blood flow information of a lesion are all well demonstrated by OCTA. Third, the distinctively delineated lesion margin benefits the quantification of neovascularization and nonperfusion areas that may be obscured by diffuse leakage in FA or ICGA [16]. Fourth, quantitative measurements of neovascularization in retina or choroid will be possible if co-developed software is added to the Optovue. In comparison, the invasive nature of FA and ICGA hampers their application on certain patient. Moreover, they do not allow separate evaluation of different vascular plexuses that may be affected inconsistently in certain diseases. In addition, the examination process of conventional angiography is time consuming. Therefore, although ICGA and FA have commonly been used to assess the choroidal and retinal circulation respectively and are irreplaceable in detecting fundus diseases, the noninvasive OCTA is a more attractive method for frequent assessments of the posterior choroid and retina in the follow-up visits and under certain situations.

OCTA owed its outstanding performances to SSADA and segmentation algorithms, which can display any plane of interest. Thus, the nonperfusion area of the superficial the deep plexuses could be viewed separately, and the exact thickness of neovascularization lesion could be defined. Besides the OCTA, the RTVue XR 100 Avanti OCT has other modalities, which may assist the diagnosis of retinal and choroidal diseases. The structural OCT with enhanced depth imaging (EDI) can be realized by running the cross-line scanning pattern. It provides cross-sectional anatomic information of retina and allows measurement of choroid depth, although the image resolution acquired via this modality is not as high as that of HRA 2. When OCTA was combined with cross-sectional OCT, a more comprehensive picture of the retinal and choroidal disease was emerged. For instance, OCTA that detected CNV network, along with the B scan that elucidated the positional relation between CNV and RPE, could distinguish subtypes of CNV without the help of FA or ICGA. An angiogram showing BVN and polyps together with a simultaneous B scan showing dome-like elevation of RPE may offer a tentative diagnosis of PCV. The multi-modality may help ophthalmologists to generate prompt diagnosis of retinal and choroidal diseases.

Although OCTA images of most patients studied were of high quality, visualizing the retinal and choroidal vasculatures with high resolution and great details, OCTA also has certain limitations. First, it could not get good OCTA images of patients with poor fixation due to seriously impaired vision or nystagmus. Second, very thick hemorrhage and severe edema would make the retina unevenly thickened or elevated, interrupting the projection of the retinal vasculature onto a flat plane. Second, unlike FA, which provides dynamic and current information, the OCT angiography only yields static images, failing to show pooling, staining and leakage. Therefore, it may not provide useful information in uveitis and choroiditis and may not differentiate some active or inactive CNV lesions [17]. Third, due to the limited scope of OCTA (no more than 8 × 8 mm if montage images are not used), its application in detecting signs in periphery fundus was impaired. Fourth, media opacity, dry eyes, and poor fixation can weaken the signals. Therefore, the technique requires patient selection and careful operator technique [18].

In our study, OCTA showed strikingly similar images of neovascularization and BVN as those showed by FA and ICGA. In addition, OCTA even offered a better delineation of fovea avascular zone (FAZ) and capillary dropout. Although the hyperfluorescence in FA/ICGA corresponded well with the hyperreflection in OCTA, the polypoidal lesions that were hyperfluorescent on ICGA appeared hyperreflective, medium or hyporeflective when scrolling through different depths from RPE to choroid capillary in OCTA. This was in accordance with another study, in which the polypoidal lesion appeared hyporeflective at the choroid capillary level and hyperreflective in a more anterior plane [19].

## Conclusions

In summary, OCTA provided a better projection of vascular pathologies of the posterior retina and choroid and could determine the precise location of the vascular lesion. OCTA is a reliable, reproducible, noninvasive, and time-saving new technology, which could result in a better patient compliance. OCTA is a promising tool for clinicians to make preliminary diagnosis and assess treatment efficacy in the follow-up visits.

## Abbreviations

AMD, age-related macular degeneration; BRVO, branch retinal vein occlusion; BVN, branching vascular network; CNV, choroidal neovascularization; FA, fluorescein angiography; FAZ, foveal avascular zone; ICGA, indocyanine green angiography; MCT, motion correction technology; NPDR, non-proliferative diabetic retinopathy; OCTA, optical coherence tomography angiography; PCV, polypoidal choroidal vasculopathy; PDR, proliferative diabetic retinopathy; RPE, retinal pigment epithelium; SSADA, split-spectrum amplitude-decorrelation angiography.
